# Supplementary material for: Characterization of linear epitope specificity of antibodies potentially contributing to spontaneous clearance of hepatitis C virus
Source: PLoS One. 2021 Aug 27;16(8):e0256816. doi: 10.1371/journal.pone.0256816 (PMC8396737; doi:10.1371/journal.pone.0256816)
Supplement: S1 Fig — The alignment shows significant amino acid sequence conservation in these regions among different genotypes. (PDF) [file pone.0256816.s001.pdf]

HCV Genotypes	aa412–423	aa432–443	aa523–532
1a	QLINTNGSWHIN	SLNTGWLAGLFY	GAPTYSWGAN
1b	QLVNTNGSWHIN	SLNTGFIASLFY	GAPTYTWGAN
2a	QLINTNGSWHIN	SLNTGFIASLFY	GAPTYTWGEN
2b	QLINTNGSWHIN	SLNTGFIASLFY	GVPTYSWGEN
3a	QLVNTNGSWHIN	SLNTGFIAGLFY	GAPTYSWGAN
4a	QLINTNGSWHIN	SLNTGFLASLFY	GVPTYTWGEN
4g	QLINTNGSWHIN	SLNTGFIAGLFY	GAPTYSWGEN
5	QFVNTNGSWHIN	SLQTGFIAGLMY	GYPTYNWGEN
6a	QLVNTNGSWHIN	SLNTGFIASLFY	GNPTYTWGEN
7	QLINTNGSWHIN	SLQTGFIAALFY	GVPTYTWGEN
	*::*********	**:**::*.*:*	*.***.**:*

S1 Fig. Alignment of amino acid sequences of three linear epitopes from different HCV genotypes. The alignment shows significant amino acid sequence conservation in these regions among different genotypes
