# Supplementary material for: Characterization of linear epitope specificity of antibodies potentially contributing to spontaneous clearance of hepatitis C virus
Source: PLoS One. 2021 Aug 27;16(8):e0256816. doi: 10.1371/journal.pone.0256816 (PMC8396737; doi:10.1371/journal.pone.0256816)
Supplement: S2 Fig — The data showing no viral neutralization by any of the serum samples at 10-fold dilution. Only the sample 226 showed slight activity. (PDF) [file pone.0256816.s002.pdf]

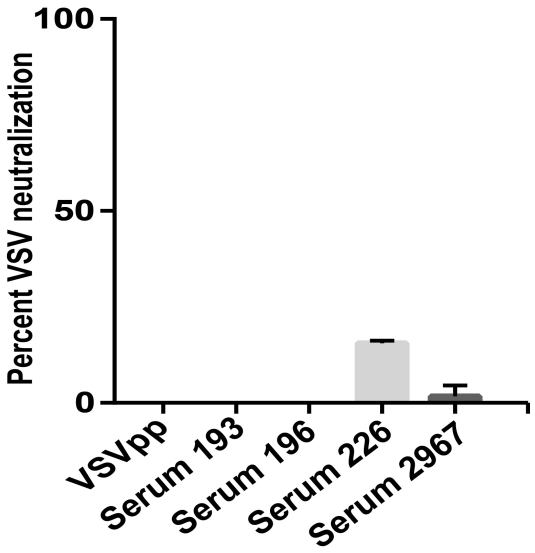

S2 Fig. VSV neutralization by four different human serum samples. The data showing no viral neutralization by any of the serum samples at 10-fold dilution. Only the sample 226 showed slight activity.
