# Supplementary material for: Characterization of linear epitope specificity of antibodies potentially contributing to spontaneous clearance of hepatitis C virus
Source: PLoS One. 2021 Aug 27;16(8):e0256816. doi: 10.1371/journal.pone.0256816 (PMC8396737; doi:10.1371/journal.pone.0256816)
Supplement: Attachment — Submitted filename: Response to reviewers.docx [file pone.0256816.s004.docx]

### June 24, 2021

### The Editor-in-Chief

Plos-One

**Re: Response to reviewers**

Dear Dr Sripathi M Sureban,

Thank you very much for the review of our manuscript titled “Characterization of epitope specificity of antibodies potentially contributing to spontaneous clearance of hepatitis C virus”. We deeply appreciate the efforts of reviewers for careful reading of the manuscript and providing insightful comments towards improving the manuscript. The manuscript has now been revised in light of reviewer’s comments and response to each comment has been included below.

ACADEMIC EDITOR: I commend the team for such a extensive study and is need of the hour. Please respond with comments (point-by-point) to both the reviewers concerns. Reviewer 1 has raised very important comments and is necessary to fulfill these comments.

Response: Thank you very much for the encouraging comments and appraising the efforts to conduct this study. We have addressed all these comments in particular the comments of reviewer 1.

Reviewer #1: Ahsen and colleagues describe an analysis of the epitope specificity of the polyclonal antibody response in patients who spontaneously resolve hepatitis C virus infection, comparing with the antibody response in individuals who progress to chronic infection. The study addresses an important question, as the quality of the antibody response associated with protection is useful for informing vaccine design. However, I have the following concerns with the design and execution of the study:

Major points

1.The design of the study only permits interrogation of the antibody response to linear peptides. As the authors indicate in the Discussion section, it is well established that the majority of neutralizing antibodies isolated from infected humans recognise conformation-dependent epitopes. The approaches used in this study only examine reactivity to three short peptide epitopes, and as such there is the possibility of lack of interrogation of important antibodies binding to conformation-dependent epitopes in these peptide regions on the surface of the E2 protein.

Response: Thank you very much for the encouraging comments and appraising the efforts. This is true that majority of HCV neutralizing antibodies are conformational-epitope specific. However, linear epitope specific antibodies have also been shown to neutralize a broad range of circulating HCV strains. The well studied examples are human mAb HCV1 (*Kong L, et al. Proc Natl Acad Sci U S A. 2012, 109(24):9499-504; and Morin TJ, et al. PLoS Pathog. 2012;8(8):e1002895*) and mouse mAb AP33, and many others. There are different hypotheses about the spontaneous HCV clearance suggesting the role of cellular immunity and humoral response including linear epitope specific antibodies. Complete investigation on the role of every component of humoral immunity is beyond the scope of this study. Our focus in this study is to characterize the potential role of only linear epitope in spontaneous clearance. The scope was possibly not very clearly demonstrated. Now to make it more clear we have added a word “linear” epitope in the title and described in the background paragraph of the abstract. The significance of linear epitope was discussed in the last 7-8 lines of the introduction part.

2.The three epitope regions interrogated in the study are all variable to different degrees between different HCV infections. The infecting genotype of HCV influences this sequence variability. While genotype 3 infections are mentioned in the Results, there is no detailed information provided in the Methods about the genotypes of the infecting viruses, either in chronic infections, on in spontaneously resolving infections. Were all of these infections genotype 3 HCV? There also appears to be inconsistencies in the sequences of the selected sequences used for the study. For example, in peptide 412, the sequence RRQLVNTNGSWHINRR is used as the target peptide, but the sequence for genotype 3a indicated in supplementary material is QLINTNGSWHIN. It appears that the peptides were generated as consensus amino acid sequences of all 7 major genotypes of HCV for target peptides. Peptides representing the sequences of genotype 3 strains (if they are the main genotype infecting the study cohort) would be much better for this study. It should also be appreciated that some of the infected individuals will be infected with strains that differ in their amino acid sequence in these critical epitopes, which may influence the reactivity to the selected peptides.

Response: Thank you very much for highlighting this important point that was not clear. The main target population in the study was spontaneous resolvers. They were identified as spontaneous resolvers through screening of patients visiting primary care and gastroenterology clinics of Shalamar Hospital and PKLI by performing ELISA based diagnostic Anti-HCV antibody test followed by PCR for viral load, in case of positive ELISA test. The individuals with positive ELISA and negative PCR were shortlisted and included in the study after written consents. Their diagnostic tests were performed again after four weeks and then blood samples were collected. None of these individuals was aware of his/her past HCV infection that had been cleared without pursuing treatment. Therefore, finding genotype of the virus that had already been cleared from the patient was impossible. This is one of the biggest challenges to develop cohort of acutely infected individuals leaving them without treatment for several months or even years to study the progress of the disease and immune response at different time points. Since, the genotype information of the main population, spontaneous resolvers were impossible, we did not include the genotype factor of chronic patients as well to remain consistent. Therefore, the synthesized peptide had the consensus sequence of all major genotypes, though these peptides correspond to the most conserved regions of the E2 glycoprotein. The HCVpp containing envelope of genotype-1a was used in neutralization assays, as the assay with genotype-1a is the most sensitive. On the basis of high level of amino acid conservation in these linear epitopes and broadly neutralizing nature of most of the known mAbs targeting these linear epitopes we could hypothesize that particular linear epitope specific antibodies in serum samples could be broadly cross reactive. Although, there could have been a small proportion of strain specific antibodies in non-gentoype-1a infected individuals that might not have neutralized Gnt1a HCVpp or a small proportion of Gnt1a specific sera might not have been competed by the peptide with consensus amino acid sequence. In the follow up study, we are planning to investigate each of the serum samples against HCVpp of all seven genotypes and compete with different genotype specific peptides to obtain clearer picture. However, the current study provides information on the potential role of linear epitope specific antibodies which are non-strain specific in spontaneous clearance. This is actually the promising for vaccine design perspective. This information has been incorporated in the methods section and the sequence alignment figure has been updated after removing the terminal residues that were added just to enhance the solubility but were not the part viral sequence. Also, a small error in the numbering of amino acids in the linear epitope has been removed and reassigned the numbering according the sequence of HCV E2.

3. Line 88: The synthesis format of the peptides is not described, and it is not clear if the peptides are linear, or branched. Given that the peptides are directly coated onto the assay plates, it is possible that the antibody binding epitopes are not accessible to the antibodies present in the patient samples in this assay format. The authors must comment on the format of the assays used for these analyses. There is the potential for Type II error in the data if the peptides were linear in nature, rather than branched.

Response: The peptides used were linear as in nature they do not exist as branched. Theoretically, there are chances that a small proportion of reactive serum samples could have been missed due to the inaccessibility of the epitope in ELISA, however, the peptide ELISA technique is very well established and is vastly used for such kind of reactivity detection. Moreover, possible false positive effects were ruled out by using two types of controls, the one where irrelevant protein was coated and the second where sera from uninfected healthy individuals were used for reactivity to the three peptides. Also, the viral neutralization experiments in the next step further confirmed the reactivity observed in ELISA.

4. Line 93: antibody AP33 is indicated to be a positive control for these assays. However, AP33 is a mouse monoclonal antibody, rather than a human antibody, and as such the assay described would not work with the indicated secondary antibody. Did the authors use a humanised version of AP33? If so, this information must be provided, with an appropriate citation.

Response: Thank you very much for highlighting this, the AP33 antibody was mouse monoclonal antibody and the secondary antibody used to detect was anti-mouse. This information has been corrected in the relevant methods section.

5. Line 107: The authors should provide information on the strain used for the HCV E1/E2 envelope glycoprotein expression construct. This is extremely important; it is not clear if the neutralization assays were performed with a genotype-matched construct, or if the sequence of the E1/E2 construct matched the amino acid sequence of the peptides used in the ELISA assays.

Response: The HCVpp E1E2 envelope corresponded to genotype-1a. This information has been incorporated in the relevant section and its reason has been explained above, see the response to Comment 2.

6. The peptides used in these assays may adopt different conformations depending on the assay format. There is extensive evidence that the peptide representing epitope 412 adopts different conformational configurations, and it is highly likely that in the ELISA format used the peptide conformations will be constrained, while the soluble peptide used for competition assays may be able to adopt different conformations. The authors should comment on this potential issue comparing datasets.

Response: This is well known that most of the peptides when bound to their respective proteins or receptor they adopt specific structural conformation, but not just the peptide 412. Certainly, the surface bound peptides, whether in ELISA setup or in vastly used biophysical methods such as surface plasmon resonance based Biacore or Bio-layer interferometry based system to detect binding, exhibit conformational constraints as compared to free peptide in solution. However, all these experimental techniques are widely used to detect binding despite binding affinity might have been compromised due to lack of optimum conformation. For structural and conformation information such experimental techniques are not used. In this particular study, ELISA was used just to detect the reactivity, which might not have resulted from optimum binding affinity due to constraint conformational space of the peptide. While in the neutralization assay, the observed effect could be resulting from optimum affinity as the peptide could adopt bioactive structural conformation.

7. Figure 1: the authors should present the ELISA binding data from the uninfected 'healthy' controls in this as an additional panel for each peptide. It is not appropriate for the background cut-off to be set at the mean value of reactivity of six healthy control sera. It would be more appropriate to set the cut-off value at either twice the mean of the negative controls, or mean of controls +2x the standard deviation (if the data fit a Gaussian distribution). With the cut-off values set as presented, many of the ‘positive’ results are likely to be similar to those from the control dataset.

Response: The Figure 1 has been updated by incorporating the panel C related to ELISA binding data from the uninfected healthy controls and also corrected the information about the cut-off value which was already the mean of controls + 3x the standard deviation, but had not described correctly in the figure caption previously. Now it has been corrected.

8. Figure 4: the authors describe the use of VSV-G pseudotyped viruses as a control. They should present the data demonstrating that neutralization by serum samples was specific to HCV. Also, healthy, HCV-negative control sera should also be included in the datasets describing neutralization, to demonstrate that the observed neutralization was due to anti-HCV antibodies.

Response: A figure demonstrating the VSV-G pseudotyped virus neutralizing effect of five different serum samples has been provided as Figure S2 (supplementary material). We tested five different serum samples against VSVpp at the 10-fold dilution of the sera that was the higher concentration in HCVpp experiments. At this higher concentration there was negligible neutralizing effect, therefore we did not perform dose response curve experiments and placed this data in a separate figure (S2 Fig). Also, the effect of sera from five healthy individuals was tested against HCVpp at single concentration (10-fold dilution); due to the absence of HCV neutralization at this higher concentration, the dose response curve experiments were not performed. This has been explained now in the results, first paragraph under the title “HCV neutralization”.

9. Figure 5: The authors should include monoclonal neutralizing antibodies recognising peptides 412 and 434 in these assays to demonstrate that the consensus peptides have the potential to inhibit specific neutralization in each case. I appreciate that neutralizing mAbs recognising peptide 524 may not be available. Even if a mAb for peptide 434 is unavailable, they could perform this experiment for the combination of mAb AP33 and peptide 412.

Response: The experiment with mAb AP33 was performed in the absence and presence of peptide 412, and has been incorporated in Fig5 also describing in the figure caption. The 412 peptide clearly demonstrates the competition with the mAb.

Minor points

‘Self-resolvers’ should be more accurately referred to as ‘spontaneous resolvers’

Response: Corrected, thanks for highlighting this

Lines 166-171: It is not clear why the figure legend for Figure 1 is presented here.

Response: This is according to the author’s instructions that state “Figure captions are inserted immediately after the first paragraph in which the figure is cited. Figure files are uploaded separately”.

Lines 187-188: it is not clear why the figure legend for Figure 2 is presented here.

Response: This is according to the author’s instructions that state “Figure captions are inserted immediately after the first paragraph in which the figure is cited. Figure files are uploaded separately”.

Lines 208-214: it is not clear why the figure legend for Figure 3 is presented here.

Response: This is according to the author’s instructions that state “Figure captions are inserted immediately after the first paragraph in which the figure is cited. Figure files are uploaded separately”.

Lines 226-232: it is not clear why the figure legend for Figure 4 is presented here.

Response: This is according to the author’s instructions that state “Figure captions are inserted immediately after the first paragraph in which the figure is cited. Figure files are uploaded separately”.

Figure 4: The third panel on the first row indicates that neutralization is tending to plateau at ~65% neutralization, and at the greatest dilution is trending to a minimum neutralization of ~15%. These data make no biological sense, and are likely to be artefacts of the way the data has been analysed using Graphpad Prism.

Response: The two plots have been corrected that had mistakenly incorporated double data for some dilutions.

Figure 4: the X axis on all panels is labelled as ‘log of serum dilution’, but no logarithmic base is indicated. Presumably they are base 10, but this needs to be indicated.

Response: It has been corrected in all figures as log10.

Line 367: ‘Zhen-Young K et al’ should read ‘Keck et al’

Response: Corrected, thanks

Reviewer #2: This manuscript has the potential to be accepted, but there are a few key problems that need to be explained or rectified before we can move forward and take constructive action. Methods and discussion requires improvement.

General comments:

HCV infection will almost certainly be eradicated globally if a preventive HCV vaccination is developed. The mechanisms of effective HCV-specific immunity in acute-resolving HCV infection, as well as the mechanisms of HCV-specific CD8+ T cell failure in persistent infection, have made significant progress in recent years. A future effective vaccination will almost certainly need to produce both cell-mediated and humoral immunity. Cross-reactive epitopes, conserved areas within the HCV genome, correlates of protective immunity, and the role of bnAbs are all critically needed to attain this goal. I commend the author for conducting this research work, which is urgently needed. This manuscript has the potential to be accepted, but there are a few key problems that need to be explained or rectified before we can move forward and take constructive action.

**Major comments:**

1. Page No.3, Line no.75 - I would recommend that the authors describe the manuscript's aims and hypothesis rather than the results in the final paragraph of the introduction.

Response: The findings and the results discussed in the introduction sections are from the previous work of different research groups on the basis of what the current study was designed. However, we have tried to make it more clear incorporating clear hypothesis and aim of the current study. The last sentence of the introduction is the statement about what we have done over all in this study.

2. Page No.4, Line no. 83 – How the sample size (n = 84) was calculated and segregated as chronic patients (n = 30), Spontaneous resolver (n = 49) and healthy donors (n = 5).

Response: The main target population was the spontaneous resolvers. We aimed to include as many samples as possible from this group. It took us over two years to collect 50 serum samples in collaboration with two hospitals. One of the 50 samples was wasted during handling. The sample size 49 was not the target number but the significant number we could manage in a reasonable time. The chronic patient group was used just for comparison. The healthy individual samples was used just as the experimental control but not to study a certain factor at population level that is why sample size 5 was good enough for the experimental control.

3. Page No.4, Line no. 87 - The authors has not mentioned reference for the Elisa Methodology used. If the ELISA is a standardized method, it should be mentioned.

Response: The reference has been given now

4. Page No.6, Line no. 124 – There is no reference for the peptide competition assay used in the methodology.

Response: The reference has been given now

5. Page No.7, Line No.149 – The author has defined spontaneous resolvers included in the study, but there is no defined mention of chronic patients. How the chronic patients were defined for enrolment. And also there is no data regarding the genotypes of the chronic patients enrolled in this study.

Response: The definition of chronic patients has been added in the relevant portion in the method section and definition statement of spontaneous resolvers has also been moved to the Methods section. As far as the question about genotype information is concerned, the main target population in this study was spontaneous resolvers. They were identified as spontaneous resolvers through screening of patients visiting primary care and gastroenterology clinics of Shalamar Hospital and PKLI by performing ELISA based diagnostic Anti-HCV antibody test followed by PCR for viral load, in case of positive ELISA test. The individuals with positive ELISA and negative PCR were characterized as spontaneous-resolvers. Interestingly, none of these individuals was aware of his/her past HCV infection that had been cleared without pursuing treatment. Therefore, finding genotype of the virus that had already been cleared from the patient was impossible. Since, the genotype information of the main population, spontaneous resolvers were impossible, we did not include the genotype factor of chronic patients as well to remain consistent.

6. Page No. 10, Line no. 223 – Any reason for the particular sample (SH503) which was not reactive to any of the three peptides used.

Response: As we observed, in spontaneous resolvers less than 50% of the serum samples showed reactivity in ELISA to any of the three peptides, that indicates all spontaneous resolvers may not have produced antibodies against these linear epitopes or the antibody titer is too low to be detected. The sample SH503 neutralized the virus, which suggests viral clearance is not only by the linear epitope targeted antibodies but conformational epitopes specific antibodies also significantly contribute in viral clearance. The focus of the current study is jus to analyze potential role of linear epitope targeted antibodies in spontaneous viral clearance.

7. Discussion has to be rewritten in an organized way with suitable references for proper understanding.

Response: Thanks for your help in improving this manuscript, we have improved the discussion part.

**Minor Comments:**

1. Page No.8, Line no. 176 – typographical error - it should be 10 – fold instead of 10 flod .

Response: Corrected, thanks

2. References should be in the prescribed journal format.

Response: Corrected, thanks

3. There is no data on healthy donors (n = 5) who were included in this study.
4. Response: The information has been incorporated in the relevant methods section

With very best regards

Syed Shahzad ul Hussan

Department of Biology, Lahore
University of Management Sciences
(LUMS), Lahore 54792, Pakistan
