# Supplementary material for: Characterization of linear epitope specificity of antibodies potentially contributing to spontaneous clearance of hepatitis C virus
Source: PLoS One. 2021 Aug 27;16(8):e0256816. doi: 10.1371/journal.pone.0256816 (PMC8396737; doi:10.1371/journal.pone.0256816)
Supplement: Attachment — Submitted filename: Response to reviewers.docx [file pone.0256816.s005.docx]

### July 30, 2021

### The Editor-in-Chief

Plos-One

**Re: Response to reviewers**

Dear Dr Sripathi M Sureban,

Thank you very much for the review of our manuscript titled “Characterization of linear epitope specificity of antibodies potentially contributing to spontaneous clearance of hepatitis C virus”. We deeply appreciate the efforts of reviewers for careful reading of the manuscript and providing insightful comments towards improving the manuscript. The manuscript has now been revised again in light of reviewer’s comments and response to each comment has been included below.

ACADEMIC EDITOR: The manuscript has been extensively improved from its initial version. Reviewer 2 has accepted the manuscript, however, Reviewer 1 has some minor concerns that need to be clarified before considering to accept the manuscript. Please edit and provided point-to-point response to the questions raised

Response: Thank you very much for the encouraging comments and appraising the efforts of improving the manuscript. We are thankful to Reviewer 2 for accepting this and to Reviewer 1 for his comments and suggestion to further improve the manuscript. We have addressed all these comments bellow.

Reviewer #1: In this resubmission the authors have made significant improvements to the quality of the manuscript. This has addressed many of my original concerns, providing additional data to support their conclusions. I have the following comments that need to be addressed:

Response: Thank you very much for the encouraging comments

Comments:

1. I thank the authors for the rebuttal to my original comment about the format of the ELISA assay and the possibility for false negative results. We and others found that linear peptides derived from HCV E2 directly coated to plastic surfaces were non- reactive for some patient serum samples, but that when the same linear peptides were captured to an ELISA plate using a terminal tag, sero-reactivity could be demonstrated. An alternative solution to this issue is to use branching synthesis when making the peptides to provide additional epitope accessibility (there is no implication that the structure on the surface of E2 is branched) (see Tarr et al, Hepatology 2006 https://doi.org/10.1002/hep.21088; Tarr et al, Journal of General Virology https://doi.org/10.1099/vir.0.83065-0). The authors should mention this potential limitation in their assay format in the Discussion section. The authors cite previous studies that use this method, but it is possible that they also suffer from this limitation.

Response: According to the suggestion, we have incorporated a sentence in the second paragraph of the discussion section as “*We do want to mention that direct immobilization of linear peptides on the surface of ELISA plate has potential limitation of false negative outcome due to the conformational restriction of the immobilized peptide. Owing to this limitation, reactivity of a small proportion of antibodies might have missed in our ELISA assay*”.

2. Line 132: the authors have provided the genotype of the strain used for pseudotype generation. However, the neutralization sensitivity of different genotype 1a strains varies widely (Wasilewski, Ray & Bailey, 2016 10.1099/jgv.0.000608). The authors should provide the strain designation of the E1/E2 construct used for these experiments. Use of the H77 strain for these types of experiments is common, and it is widely appreciated that this represents a ‘neutralization-sensitive’ phenotype, that might not represent the neutralization sensitivity of other genotype 1a strains. An indication of the strain would allow the reader to make their own interpretation of the data.

Response: We have added the required information in the method section as “*The HCV genotype-1a (strain H77) envelope-expressing vector…”*

3. Line 293: reference to ‘Table 2’ should be to Table 3.

Response: I guess this is related to the sentence “Apart from two serum samples, SH511 and SH19, HCV neutralizing activity of all peptide reactive samples were competed by the presence of respective peptides (Table 2 and Fig 5)” as there is difference in line number. The information related to SR samples is given in figure 5 but not in any table, we have removed “Table 2 reference” here.

Lines 372-373: It is plausible that the lack of neutralizing response in spontaneous resolvers could be due to the waning antibody response following lack of antigenic stimulation of B cells in these individuals in the period following clearance of the virus. Without a previous RNA-positive test, it is impossible to predict when the spontaneous resolution occurred in these individuals. I appreciate that these patients were identified as being antibody positive in clinical assays, but it would be useful to describe in the Discussion if the signals in these assays were comparable to that achieved in chronic infections, or if the antibody binding signals were low.

Response: The diagnostic anti-HCV antibody response was used qualitatively to find if the person has ever been infected with HCV. The information was collected at two different hospitals. Since, the primary purpose was to obtain qualitative information, the quantitative data of all patients was not saved, unfortunately, which could be used to determine if the difference is statistically significance in the two groups. However, quantitative data of few of the individuals were available according to which the average antibody response in spontaneous resolvers were slightly less but may not be significantly different. Therefore, we have added a brief line in the discussion section mentioning that the anti-HCV antibody response in the two groups was observed to be comparable.

5. Figure S1: The authors should revise this figure to represent the most frequent sequences occurring in each genotype (or subgenotype). For instance, for genotype 2b viruses, for sequences deposited on Genbank the sequence for the region 412-423 is rarely ‘QLVNTNGSWHIN’, and is more commonly ‘QLINTNGSWHIN’ of ‘SLINTNGSWHIN’. This impacts on the statement on Line 164 that the selected peptide represented a consensus of all major genotypes of HCV

Response: The figure has been revised according to the suggestion by incorporating most frequent sequences of different genotypes in the figure.

6. The statistical analysis for Figure 5 used repeated T tests, but there is no indication that a correction for repeated measures was performed. A one-way ANOVA with Bonferroni correction may be more appropriate where multiple comparisons are performed.

Response: We have performed the statistical analysis again according to the suggestions, updated the writing in the methods section and also redefined significance level in Figure 5.

Moreover, there was a typo (healthy was written as healty) in figure 1 that has been corrected now.

With very best regards

Syed Shahzad ul Hussan

Department of Biology, Lahore
University of Management Sciences
(LUMS), Lahore 54792, Pakistan
