# Supplementary material for: Characterization of linear epitope specificity of antibodies potentially contributing to spontaneous clearance of hepatitis C virus
Source: PLoS One. 2021 Aug 27;16(8):e0256816. doi: 10.1371/journal.pone.0256816 (PMC8396737; doi:10.1371/journal.pone.0256816)
Supplement: Attachment — Submitted filename: REVIEWER COMMENTS.docx [file pone.0256816.s003.docx]

**Comments:**

**TITLE OF THE MANUSCRIPT**: Characterization of epitope specificity of antibodies potentially contributing to spontaneous clearance of hepatitis C virus

AUTHORS:

Syed Shahzad-ul-Hussan, PhD

Asma Ahsan

Saira Dar

Fareeha Hassan

Farkhanda Ghafoor

Haroon Yousuf

General comments:

HCV infection will almost certainly be eradicated globally if a preventive HCV vaccination is developed. The mechanisms of effective HCV-specific immunity in acute-resolving HCV infection, as well as the mechanisms of HCV-specific CD8+ T cell failure in persistent infection, have made significant progress in recent years. A future effective vaccination will almost certainly need to produce both cell-mediated and humoral immunity. Cross-reactive epitopes, conserved areas within the HCV genome, correlates of protective immunity, and the role of bnAbs are all critically needed to attain this goal. I commend the author for conducting this research work, which is urgently needed. This manuscript has the potential to be accepted, but there are a few key problems that need to be explained or rectified before we can move forward and take constructive action.

**Major comments:**

1. Page No.3, Line no.75 - I would recommend that the authors describe the manuscript's aims and hypothesis rather than the results in the final paragraph of the introduction.
2. Page No.4, Line no. 83 – How the sample size (n = 84) was calculated and segregated as chronic patients (n = 30), Spontaneous resolver (n = 49) and healthy donors (n = 5).
3. Page No.4, Line no. 87 - The authors has not mentioned reference for the Elisa Methodology used. If the ELISA is a standardized method, it should be mentioned.
4. Page No.6, Line no. 124 – There is no reference for the peptide competition assay used in the methodology.
5. Page No.7, Line No.149 – The author has defined spontaneous resolvers included in the study, but there is no defined mention of chronic patients. How the chronic patients were defined for enrolment. And also there is no data regarding the genotypes of the chronic patients enrolled in this study.
6. Page No. 10, Line no. 223 – Any reason for the particular sample (SH503) which was not reactive to any of the three peptides used.
7. Discussion has to be rewritten in an organized way with suitable references for proper understanding.

**Minor Comments:**

1. Page No.8, Line no. 176 – typographical error - it should be 10 – fold instead of 10 flod .
2. References should be in the prescribed journal format.
3. There is no data on healthy donors (n = 5) who were included in this study.
